# Fetal growth at term and placental oxidative stress in a tissue micro-array model: a histological and immunohistochemistry study

**DOI:** 10.1007/s00418-023-02212-6

**Published:** 2023-06-12

**Authors:** Serena Xodo, Lisa Celante, Stefania Liviero, Maria Orsaria, Laura Mariuzzi, Matteo De Luca, Giuseppe Damante, Lorenza Driul, Angelo Cagnacci, Annalisa Ferino, Eros Di Giorgio, Luigi Xodo, Ambrogio Pietro Londero

**Affiliations:** 1 Clinic of Obstetrics and Gynecology, DAME, University of Udine, 33100 Udine, Italy; 2 Institute of Pathology, Academic Hospital “Azienda Sanitaria Universitaria Integrata di Udine”, 33100 Udine, Italy; 3 Institute of Pathology, DAME, University of Udine, 33100 Udine, Italy; 4 Institute of Medical Genetics, Academic Hospital “Azienda Sanitaria Universitaria Integrata di Udine”, DAME, University of Udine, 33100 Udine, Italy; 5 Department of Neuroscience, Rehabilitation, Ophtalmology, Genetics, Maternal and Infant Health, University of Genoa, Largo Rosanna Benzi 10, 16132 Genova, Italy; 6 Laboratory of Biochemistry, Department of Medicine, University of Udine, 33100 Udine, Italy; 7 Obstetrics and Gynecology Unit, IRCCS Istituto Giannina Gaslini, 16147 Genova, GE Italy

**Keywords:** Oxidative stress, Fetal growth, Placenta, 8-Hydroxy-20deoxyguanosine, Placental histology, Sex-specific regulation

## Abstract

This study examines 8-hydroxyguanine (8-oxo-Gua) staining in placental tissue samples based on fetal size at birth as well as its relationships with placental histology and other pregnancy variables. This prospective cohort study included women > 18 years with a singleton pregnancy, a live fetus, fluency in Italian, and delivery at term. A total of 165 pregnancies were included in the study. The nuclear syncytiotrophoblast 8-oxo-Gua staining score in LGA was substantially greater than in late FGR (*p* < 0.05), although the cytoplasm score was lower in SGA and LGA than in AGA (*p* < 0.05). Furthermore, a sex-specific pattern of 8-oxo-Gua staining was discovered in single-term placentas, with more oxidative damage found in the nuclei of syncytiotrophoblast cells and stromal and endothelial cells in AGA males compared to AGA females (*p* < 0.05). Second, the histological pattern of late FGR placentae differed by gender. Finally, a significant correlation (*p* < 0.05) was found between high-intensity 8-oxo-Gua staining in the cytoplasm of syncytiotrophoblast cells and thrombi in the chorionic plate or villi in males. On the other hand, female fetuses demonstrated a significant connection (*p* < 0.05) between high-intensity 8-oxo-Gua staining in endothelial and stromal cells and high birthweight MoM values. Our findings indicated a significant variation in the oxidative stress pattern between male and female placentae, implying that fetal growth is regulated differently in the two sexes.

**Supplementary Information:**

The online version contains supplementary material available at 10.1007/s00418-023-02212-6.

## Introduction

The negative immediate and long-term perinatal consequences make fetal growth restriction (FGR) one of the most researched topics in feto-maternal medicine (Miller et al. 2016). Fetal growth restriction is described as the failure of the fetus to reach its genetically set growth potential, which is caused mainly by insufficient remodeling of the uterine spiral arteries that supply the placenta during pregnancy. The subsequent malperfusion causes cell stress inside the placental tissues, reducing nutrition transport to the fetus (Burton and Jauniaux 2018). Recent research has found that not only FGR but also small-for-gestational-age (SGA) fetuses have suboptimal neurodevelopment and an increased cardiovascular risk later in life, implying that SGA is a milder form of FGR rather than a group of smaller normal fetuses (Varley et al. 2023; Vollmer and Edmonds 2019). Based on this evidence, several authors hypothesized that less severe abnormalities in vascular remodeling result in pregnancies with variable degrees of fetal impairment, which correlate to varying degrees of fetal smallness (Burton and Jauniaux 2004, 2018).

Malperfusion of any organ, including the placenta, potently induces oxidative stress (Burton and Jauniaux 2011; Hubel 1999). Maternal vascular malperfusion (MVM) is the histopathological result of the failure of extravillous trophoblasts to implant correctly in the decidua and remodel the spiral arteries in early pregnancy (Redline 2015; Redline et al. 2004). Decidual arteriopathy, rapid villous maturation, villous infarction, and abruptio placentae are the hallmarks of MVM. Stillbirth, fetal death, FGR, and premature delivery are common complications of MVM (Redline 2015; Stallmach et al. 2001). Fetal vascular malperfusion (FMV) is a well-known cause of stillbirth, fetal mortality, and central nervous system injury. Despite its multifactorial etiology, obstructed umbilical blood flow is the most common cause of FVM (Redline and Ravishankar 2018). Luminal thrombi, changes to the wall of the major fetal arteries, and a considerable number of downstream avascular villi are histopathological observations in FVM (Redline and Pappin 1995). The syncytiotrophoblast is the epithelial layer that covers the placental villous tree; it is generated by the continuous fusion of underlying cytotrophoblast cells (Mayhew and Simpson 1994). Syncytiotrophoblast cells are susceptible to oxidative stress because of their close association with maternal blood and limited antioxidant activity (Cadenas and Davies 2000; Fogarty et al. 2013; Watson et al. 1998). Oxidative stress occurs when the production of highly reactive oxygen species (ROS) exceeds a cell’s ability to detoxify them, causing indiscriminate damage to all biological components in the immediate surroundings, including proteins, lipids, and DNA (Yoshikawa and Naito 2002). As a result, cell function is hindered, and cell death may occur in the most severe cases. Damaged cells can be recognized using 8-oxo-Gua, a biomarker for oxidative DNA/RNA damage generated when guanine is exposed to ROS (Shigenaga and Ames 1991). However, it is unclear to what extent placental oxidative stress plays a role in the pathophysiology of fetal smallness at term. 8-Hydroxyguanine (8-oxo-Gua) is an oxidative stress biomarker (Yoshikawa and Naito 2002). Furthermore, because sex differences are recognized to contribute to altered growth and developmental outcomes, it remains to be determined whether oxidative stress affects males and females differently (Chatterjee et al. 2022).

### Objective

The main objective of this study was to evaluate the 8-oxo-Gua staining in placental tissue samples according to the different fetal growth patterns at birth. Second, we aimed to evaluate the correlations among 8-oxo-Gua, placental histology, fetal sex, and other pregnancy biochemical and clinical characteristics.

## Materials and methods

### Study design and setting

This study was a prospective cohort study conducted from May 2021 to August 2022 at the University Hospital of Udine. Women who consented to participate in the study were enrolled at the Clinic of Obstetrics and Gynecology. Fetal growth was regularly monitored through consecutive ultrasound scans, including the first-trimester screening for chromosomal abnormalities and major defects, the second-trimester screening for anatomical defects, and two growth scans after 29 weeks of gestation at least 2 weeks apart. Moreover, further ultrasound evaluations were carried out according to women’s clinical needs. After delivery, the placenta was sent to the Institute of Pathology for macroscopic and microscopic examination, tissue microarray (TMA) preparation, and immunohistochemical analysis.

The regional review board and clinical research center of the hospital approved the present study (protocol number: ASUFC/2021/0044374), which complied with the requirements of the general authorization of the Italian Data Protection Authority for scientific research purposes. Furthermore, all ethical principles of the Declaration of Helsinki were respected.

### Participants of the study

Women who attended routine ultrasound examinations during pregnancy were recruited for this prospective observational study. Inclusion criteria were age of least 18 years old, a singleton pregnancy, a live fetus at the time of the ultrasound scan, proficiency in Italian, and delivery at the term of gestation. Exclusion criteria were fetal abnormalities detected antenatally, endocrine disorders (e.g., maternal pre-pregnancy diabetes, hypothyroidism, or hyperthyroidism), maternal hypertension, pregnancy-related diseases other than isolated fetal growth disorders (e.g., gestational diabetes or cholestasis), multiple pregnancies, and severe maternal psychiatric illness.

### Variables considered

Antibody anti-8-oxo-Gua was used for immunohistochemical evaluation of placental oxidative stress. The placental examination was performed according to the Amsterdam Placental Workshop Group Consensus Statement (Khong et al. 2016). A food frequency questionnaire (FFQ) was used to assess the presence of a high-fat diet and a diet with high levels of saturated fat (Buscemi et al. 2015). At the time of FFQ, the average gestational age was 26.49 weeks (± 5.04).

Recruitment and data collection occurred after women had been informed and had given written informed consent to participate. Residents and obstetricians involved in this study collected data on maternal characteristics [i.e., age, parity, pre-pregnancy body mass index (BMI), and tobacco smoke], medical history, pregnancy complications, mode of delivery, and neonatal health.

### Definition of outcomes and variables

The international consensus statement developed by Gordijn and colleagues was adopted to define fetal smallness accurately (Gordijn et al. 2016). Fetal growth was determined according to the fetal weight Hadlock charts (Hadlock et al. 1991). Fetal abdominal circumference growth was assessed according to the Intergrowth 21 (IG-21) standard (Papageorghiou et al. 2014; Visentin et al. 2022). The estimated fetal growth at birth was then confirmed by the newborn’s birth weight for each patient in the study. Fetal Dopplers were assessed using previously published reference charts (Arduini and Rizzo 1990; Baschat and Gembruch 2003). Neonatal weights in non-FGR fetuses were categorized according to the Italian post-natal growth standards in three further groups: (1) appropriate for gestational age (AGA); (2) small for gestational age (SGA, birthweight between the 3rd and the 10th centile); (3) large for gestational age (LGA, with a birthweight > 90th centile) (Bertino et al. 2010; Salomon et al. 2019). The neonatal weight was also assessed as a multiple of the median (MoM) as previously described (Xodo et al. 2022). The neonatal weight MoM is the ratio between the observed birthweight and the 50th percentile of birthweight (sex and parity specific) at the same gestational age (Bertino et al. 2010; Xodo et al. 2022). As previously defined, placental weight in grams divided by birth weight in grams yielded the placental index (Londero et al. 2021). All gross and microscopic placental findings were defined according to the Amsterdam Placental Workshop Group Consensus Statement (Khong et al. 2016).

### Placental examination

The Amsterdam Placental Workshop Group Consensus Statement for placental tissue sampling was observed (Khong et al. 2016). The histological sampling included five blocks: one block representing a roll of extraplacental membranes taken from the rupture edge to the placental margin; one block including three cross-sections of the umbilical cord (one from the portion next to the fetal insertion, one from the intermediate portion, and one at approximately 3 cm from the placental insertion); three blocks each containing a full-thickness section of normal-appearing placental parenchyma.

### Tissue microarray (TMA) preparation

**Fig. 1:**
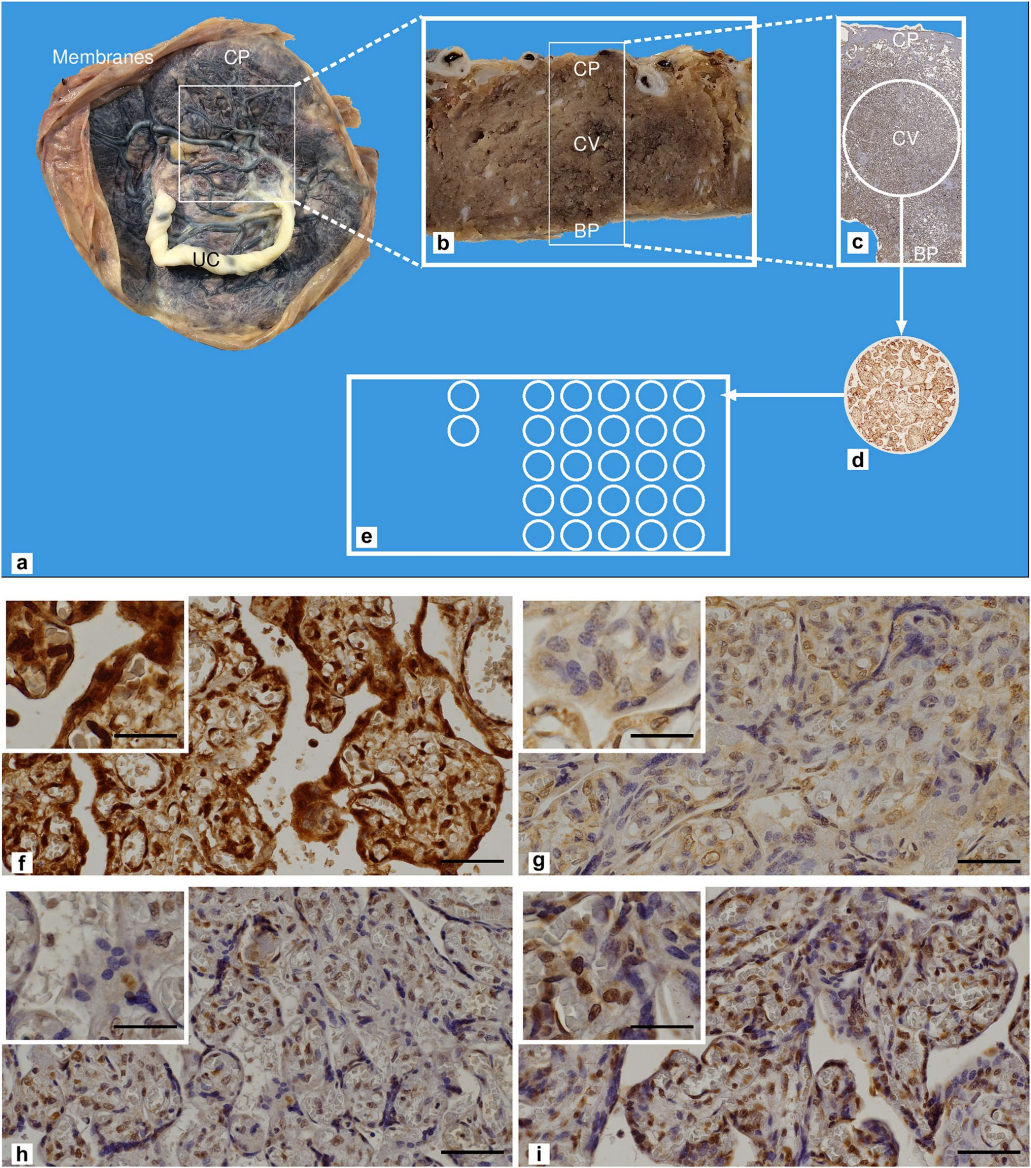
**a**, **b**, **c**, **d**, and **e** Gross and microscopic images of the placenta to demonstrate placenta sampling localization for the tissue microarray (TMA) [*UC* umbilical cord, *CP* chorionic plate, *CV* chorionic villi, *BP* basal plate (maternal side of the placenta)]. **a** Whole placenta with membranes and umbilical cord. **b** Gross figure taken from the placenta section. **c** Whole placenta thickness microscopic figure with placental 8-oxo-Gua staining. **d** TMA core showing 8-oxo-Gua staining. **e** TMA diagram. **f**, **g**, **h**, and **i** Examples of placental 8-oxo-Gua staining (× 200, scale bar length 60 μm; in the small white boxes at × 400, scale bar length 30 μm). **f** Cytoplasm and nuclear high staining score. **g** Low cytoplasm 8-oxo-Gua staining. **h** Low nuclear staining. **i** High nuclear staining

The current standard of care for pathological tissue analysis is formalin fixation and embedding in paraffin, followed by immunohistochemical staining and microscopic examination. However, a typical paraffin tissue block is exhausted after about 100 sections (Rimm et al. 2001). TMA represents a mechanism for effective tissue amplification. For the present study, sections of 4 μm thickness were obtained from samples fixed in formalin and included in paraffin. Glasses were stained with hematoxylin and eosin (EE) after section deparaffinization and rehydration, allowing pathologists to meticulously examine the tissue morphology and identify the areas for TMA preparation. The placenta was sampled twice in the central and paracentral regions on two different portions targeting the villi, as shown in Fig. 1a–e. The Beecher Instruments arraying device was used to prepare the TMA. The array construction is based on a coring system. Two hollow needles were used, one in the donor block and the other in the receiver block. First, the receiver block was perforated, and then the donor block was biopsied from specific areas previously labeled worthy of interest. The removed tissue was then carefully released into the core of the receiver block. This procedure was repeated for each tissue sample. After the array was built, the receiver block was placed facing downward on a glass at 37 °C for 15 min. This way, the paraffin melted slightly, and the cores adhered to the paraffin. Following this step, the glass was gently pushed against the receiver block to further improve the core’s adherence to paraffin. Finally, the receiver block with the glass was placed in ice, and only after cooling was the glass separated from the block. Once the recipient block was filled (as determined by the design of the individual array), it was sectioned to reveal 1.5-mm-diameter circles of tissue from each case (Fruscalzo et al. 2020).

### Immunohistochemical protocol and microscope analysis

Four-micrometer-thick sections were cut from the TMA blocks and then placed in a heater at 60 °C for 20 min to increase their adherence to the glasses. Once deparaffinized and rehydrated for antigen retrieval, the sections were incubated in Tris-EDTA buffer (Dako Target Retrieval Solution pH 9, dilution 1:10) at 98°C for 5 min. Endogenous peroxidase activity was blocked by incubation in 0.1% hydrogen peroxide in absolute methanol for 10 min at room temperature. After washing in PBS (Dulbecco’s phosphate buffer saline), sections were incubated in a humidity chamber at +4 °C overnight with anti-8-oxo-Gua antibody (JaICA, monoclonal antibody clone N45.1) diluted 1:10. The Dako REAL™ EnVision™ HRP Rabbit/Mouse was used as secondary antibody, with incubation in a whet chamber, at room temperature, 40 min long. After another washing in PBS, the peroxidase activity, which is used to mark the secondary antibody, was retrieved through incubation with the substrate Dako REAL™ DAB + chromogen (diluted 1:50) in a humidity chamber at room temperature for 5 min. Finally, after a quick washing in distilled water, the slides were counterstained with Gill hematoxylin and mounted with Histomount (National Diagnostics, NJ, USA), making them ready for evaluation. We utilized the primary antibody without the secondary antibody as a negative control. Various manufacturer-recommended tissue samples were analyzed to determine the positive control. Then, tissue slices were incubated in the absence of the primary antibody to ensure that the primary antibodies were specific.

Two observers blinded to the clinical data inspected and analyzed the stainings on a multi-head Eclipse Ni-U microscope with a ×10 ocular and ×20 or ×40 objectives (Nikon Europe B.V., Amstelveen, The Netherlands). The immunoreactivity was assessed separately in the villi’s syncytiotrophoblast, endothelial, and stromal cells. The intensity score of cytoplasm staining was graded as strong 3, moderate 2, weak 1, and absent 0 (Fruscalzo et al. 2020). H-score was used to assess nuclear staining (the product of the actual percentage of positive-stained nuclei and intensity score giving a possible range of 0–300). In case of disagreement, the two pathologists performed a joint assessment (Fruscalzo et al. 2020, 2021).

### Data analysis

The data were analyzed using the R program (version 4.2.1), and a *p* < 0.05 was considered significant. The a priori target sample size for the control group was 130 pregnancies, with a sample size of 14 cases (all 131 available controls were included). With 80% power, this sample size is large enough to detect differences in IHC scores at a 5% (two-sided) significance level. A nonparametric correction was used, and a large Cohen effect size (*d* = 0.8) was deemed relevant. The Kolmogorov-Smirnov test assessed the normality of the distribution. For non-parametric distribution, the continuous variables were described with median and interquartile range (IQR). For parametric continuous variables, the mean (± standard deviation) was used. Percentages and absolute values described the categorical variables. In addition, the following were performed where appropriate: *t*-test, one-way ANOVA, Wilcoxon test, Kruskal-Wallis test, Spearman test, chi-square test, or Fisher’s exact test.

## Results

### Population description

The median gestational age of the 165 women included at birth was 39 weeks (IQR 38-40) (Supplementary Figure 1). Supplementary Table 1 shows the remaining population characteristics. Supplementary Tables 2 and 3 illustrate the histological and immunohistochemical findings of the 165 placentae. Supplementary Table 4 shows the general characteristics of the four fetal growth groups, and the histological differences are shown in Table 1.

**Table 1:** Histology and immunohistochemical 8-hydroxyguanine (8-oxo-Gua) staining differences between fetal growth groups

	FGR (15)	SGA (9)	AGA (131)	LGA (10)	*p*
Histology					
Maternal vascular malperfusion	46.67% (7/15)	22.22% (2/9)	22.90% (30/131)	10.00% (1/10)	NS
Placental hypoplasia	26.67% (4/15)	11.11% (1/9)	7.63% (10/131)	0.00% (0/10)	2
Placental infarction	6.67% (1/15)	22.22% (2/9)	11.45% (15/131)	0.00% (0/10)	NS
Retroplacental hemorrhage	0.00% (0/15)	0.00% (0/9)	0.76% (1/131)	10.00% (1/10)	NS
Accelerated villous maturation	20.00% (3/15)	0.00% (0/9)	0.76% (1/131)	0.00% (0/10)	2
Distal villous hypoplasia	13.33% (2/15)	0.00% (0/9)	3.82% (5/131)	0.00% (0/10)	NS
Fetal vascular malperfusion	13.33% (2/15)	22.22% (2/9)	12.21% (16/131)	0.00% (0/10)	NS
Avascular villi	13.33% (2/15)	11.11% (1/9)	4.58% (6/131)	0.00% (0/10)	NS
Thrombi (chorionic plate or major stem villi)	6.67% (1/15)	11.11% (1/9)	9.16% (12/131)	0.00% (0/10)	NS
Delayed villous maturation	60.00% (9/15)	88.89% (8/9)	59.54% (78/131)	50.00% (5/10)	NS
IHC					
Nuclear staining syncytiotrophoblast (H-score)	45.00 (30.62–83.75)	105.00 (12.50–125.00)	65.00 (35.00–111.88)	100.00 (36.25–120.31)	NS
Nuclear staining syncytiotrophoblast (intensity score)	2.00 (2.00–2.00)	2.00 (1.50–2.50)	2.00 (2.00–2.50)	2.00 (2.00–2.38)	NS
Nuclear staining syncytiotrophoblast (percentage of positive nuclei)	22.50 (17.50–35.00)	52.50 (12.50–60.00)	30.00 (17.50–50.00)	46.25 (35.00–59.38)	3
Cytoplasm staining syncytiotrophoblast (intensity score)	2.00 (1.00–2.00)	0.50 (0.00–1.00)	1.50 (1.00–2.00)	1.00 (0.12–1.00)	4.6
Stromal and endothelial cells (intensity score)	2.00 (2.00–2.75)	2.00 (1.00–2.50)	2.50 (2.00–3.00)	2.50 (2.00–3.00)	NS

Differences statistically significant (*p* < 0.05): (1) FGR vs. SGA; (2) FGR vs. AGA; (3) FGR vs. LGA; (4) SGA vs. AGA; (5) SGA vs. LGA; (6) AGA vs. LGA

### 8-Oxo-Gua staining, fetal growth, and fetal sex

The percentage of 8-oxo-Gua positive nuclei of syncytiotrophoblast cells was significantly higher in LGA compared to late FGR, and the intensity score of syncytiotrophoblast cytoplasm immunostaining was significantly lower in SGA and LGA compared to AGA (Table 1 and Fig. 1f–i). Table 2 and supplementary table 5 exhibit the clinical, histologic, and immunohistochemical differences between females and males among AGA neonates. Compared to females, the male placentae had significantly higher nuclear staining in syncytiotrophoblast cells and higher intensity score staining in stromal and endothelial cells. Supplementary Table 6 shows the general characteristics of the four different fetal growth groups in female fetuses. Table 3 shows the histological and immunohistochemical differences between female fetal groups. This population consisted of 87 fetuses: 9 late FGR, 2 SGA, 72 AGA, and 4 LGA. Smaller fetuses (late FGR and SGA) appeared to have more commonly placental hypoplasia (SGA 50.0% vs. AGA 25.0%) and accelerated villous maturation (late FGR 11.11% vs. AGA 0.00%). The intensity score of the syncytiotrophoblast nuclear staining was significantly higher in SGA than in AGA (2.75, IQR 2.62–2.88 vs. 2.00, IQR 2.00–2.25), while the percentage of 8-oxo-Gua positive nuclei of syncytiotrophoblast cells was significantly higher in LGA compared to AGA (56.25, IQR 46.25-69.38 vs. 27.50, IQR 16.25-45.00). Supplementary Table 7 shows the general characteristics of the four different fetal growth groups in male fetuses. Table 4 shows the histological and immunohistochemical differences between male fetal groups. This population accounted for 78 fetuses: 6 late FGR, 7 SGA, 59 AGA, and 6 LGA. Pathological differences are shown in Table 4. Syncytiotrophoblast cytoplasm immunostaining intensity score was significantly lower in SGA and LGA than in AGA (0.00, IQR 0.00–0.75 vs. 2.00, IQR 1.00–2.00 and 0.75, IQR 0.12–1.00 vs. 2.00, IQR 1.00–2.00). Staining intensity in stromal and endothelial cells was higher in AGA than in SGA (3.00, IQR 2.00–3.00 vs. 2.00, IQR 1.00–2.00).

**Table 2:** Histology and immunohistochemical 8-hydroxyguanine (8-oxo-Gua) staining differences between fetal males and females in AGA newborns

	M (59)	F (72)	*p*
Histology			
Maternal vascular malperfusion	20.34% (12/59)	25.00% (18/72)	0.528
Placental hypoplasia	6.78% (4/59)	8.33% (6/72)	0.739
Placental infarction	11.86% (7/59)	11.11% (8/72)	0.893
Retroplacental hemorrhage	0.00% (0/59)	1.39% (1/72)	0.364
Accelerated villous maturation	1.69% (1/59)	0.00% (0/72)	0.267
Distal villous hypoplasia	3.39% (2/59)	4.17% (3/72)	0.817
Fetal vascular malperfusion	8.47% (5/59)	15.28% (11/72)	0.237
Avascular villi	3.39% (2/59)	5.56% (4/72)	0.555
Thrombi (chorionic plate or major stem villi)	6.78% (4/59)	11.11% (8/72)	0.393
Delayed villous maturation	55.93% (33/59)	62.50% (45/72)	0.446
IHC			
Nuclear staining syncytiotrophoblast (H-score)	80.00 (35.00–163.75)	60.00 (19.38–95.00)	< 0.05
Nuclear staining syncytiotrophoblast (intensity score)	2.00 (2.00–3.00)	2.00 (2.00–2.25)	< 0.05
Nuclear staining syncytiotrophoblast (percentage of positive nuclei)	35.00 (18.75–61.25)	27.50 (16.25–45.00)	< 0.05
Cytoplasm staining syncytiotrophoblast (intensity score)	2.00 (1.00–2.00)	1.50 (1.00–2.00)	0.063
Stromal and endothelial cells (intensity score)	3.00 (2.00–3.00)	2.50 (2.00–3.00)	< 0.05
Blood nucleated cells (intensity score)	1.00 (0.50–1.00)	1.00 (0.00–1.00)	0.866

**Table 3:** Histology and immunohistochemical 8-hydroxyguanine (8-oxo-Gua) staining differences between fetal growth groups

	FGR (9)	SGA (2)	AGA (72)	LGA (4)	*p*
Histology					
Maternal vascular malperfusion	33.33% (3/9)	50.00% (1/2)	25.00% (18/72)	0.00% (0/4)	NS
Placental hypoplasia	22.22% (2/9)	50.00% (1/2)	8.33% (6/72)	0.00% (0/4)	4
Placental infarction	0.00% (0/9)	50.00% (1/2)	11.11% (8/72)	0.00% (0/4)	1
Retroplacental hemorrhage	0.00% (0/9)	0.00% (0/2)	1.39% (1/72)	0.00% (0/4)	NS
Accelerated villous maturation	11.11% (1/9)	0.00% (0/2)	0.00% (0/72)	0.00% (0/4)	2
Distal villous hypoplasia	0.00% (0/9)	0.00% (0/2)	4.17% (3/72)	0.00% (0/4)	NS
Fetal vascular malperfusion	0.00% (0/9)	50.00% (1/2)	15.28% (11/72)	0.00% (0/4)	1
Avascular villi	0.00% (0/9)	0.00% (0/2)	5.56% (4/72)	0.00% (0/4)	NS
Thrombi (chorionic plate or major stem villi)	0.00% (0/9)	50.00% (1/2)	11.11% (8/72)	0.00% (0/4)	1
Delayed villous maturation	66.67% (6/9)	100.00% (2/2)	62.50% (45/72)	25.00% (1/4)	NS
IHC					
Nuclear staining syncytiotrophoblast (H-score)	40.00 (35.00–70.00)	108.12 (75.94–140.31)	60.00 (19.38–95.00)	112.50 (83.75–161.25)	NS
Nuclear staining syncytiotrophoblast (intensity score)	2.00 (2.00–2.00)	2.75 (2.62–2.88)	2.00 (2.00–2.25)	2.00 (1.75–2.25)	4
Nuclear staining syncytiotrophoblast (Percentage of positive nuclei)	20.00 (17.50–35.00)	37.50 (27.50–47.50)	27.50 (16.25–45.00)	56.25 (46.25–69.38)	6
Cytoplasm staining syncytiotrophoblast (intensity score)	2.00 (1.00–2.00)	2.00 (1.50–2.50)	1.50 (1.00–2.00)	1.00 (0.75–1.12)	NS
Stromal and endothelial cells (intensity score)	2.00 (2.00–2.00)	2.75 (2.62–2.88)	2.50 (2.00–3.00)	3.00 (2.75–3.00)	NS

In this analysis, only female fetuses were included

Differences statistically significant (*p* < 0.05): (1) FGR vs. SGA; (2) FGR vs. AGA; (3) FGR vs. LGA; (4) SGA vs. AGA; (5) SGA vs. LGA; (6) AGA vs. LGA

**Table 4:** Histology and immunohistochemical 8-hydroxyguanine (8-oxo-Gua) staining differences between fetal growth groups

	FGR (6)	SGA (7)	AGA (59)	LGA (6)	*p*
Histology					
Maternal vascular malperfusion	66.67% (4/6)	14.29% (1/7)	20.34% (12/59)	16.67% (1/6)	2
Placental hypoplasia	33.33% (2/6)	0.00% (0/7)	6.78% (4/59)	0.00% (0/6)	2
Placental infarction	16.67% (1/6)	14.29% (1/7)	11.86% (7/59)	0.00% (0/6)	NS
Retroplacental hemorrhage	0.00% (0/6)	0.00% (0/7)	0.00% (0/59)	16.67% (1/6)	6
Accelerated villous maturation	33.33% (2/6)	0.00% (0/7)	1.69% (1/59)	0.00% (0/6)	2
Distal villous hypoplasia	33.33% (2/6)	0.00% (0/7)	3.39% (2/59)	0.00% (0/6)	2
Fetal vascular malperfusion	33.33% (2/6)	14.29% (1/7)	8.47% (5/59)	0.00% (0/6)	NS
Avascular villi	33.33% (2/6)	14.29% (1/7)	3.39% (2/59)	0.00% (0/6)	2
Thrombi (chorionic plate or major stem villi)	16.67% (1/6)	0.00% (0/7)	6.78% (4/59)	0.00% (0/6)	NS
Delayed villous maturation	50.00% (3/6)	85.71% (6/7)	55.93% (33/59)	66.67% (4/6)	NS
IHC					
Nuclear staining syncytiotrophoblast (H-score)	63.12 (33.75–90.62)	105.00 (11.88–122.50)	80.00 (35.00–163.75)	70.00 (36.25–104.69)	NS
Nuclear staining syncytiotrophoblast (intensity score)	2.00 (1.62–2.38)	2.00 (1.25–2.00)	2.00 (2.00–3.00)	2.00 (2.00–2.38)	NS
Nuclear staining syncytiotrophoblast (Percentage of positive nuclei)	27.50 (18.75–34.38)	52.50 (10.00–61.25)	35.00 (18.75–61.25)	38.75 (23.75–48.12)	NS
Cytoplasm staining syncytiotrophoblast (intensity score)	1.00 (0.62–1.75)	0.00 (0.00–0.75)	2.00 (1.00–2.00)	0.75 (0.12–1.00)	4,6
Stromal and endothelial cells (intensity score)	2.25 (1.25–2.88)	2.00 (1.00–2.00)	3.00 (2.00–3.00)	2.00 (2.00–2.75)	4

In this analysis, only male fetuses were included

Differences statistically significant (*p* < 0.05): (1) FGR vs. SGA; (2) FGR vs. AGA; (3) FGR vs. LGA; (4) SGA vs. AGA; (5) SGA vs. LGA; (6) AGA vs. LGA

## Correlations

**Fig. 2:**
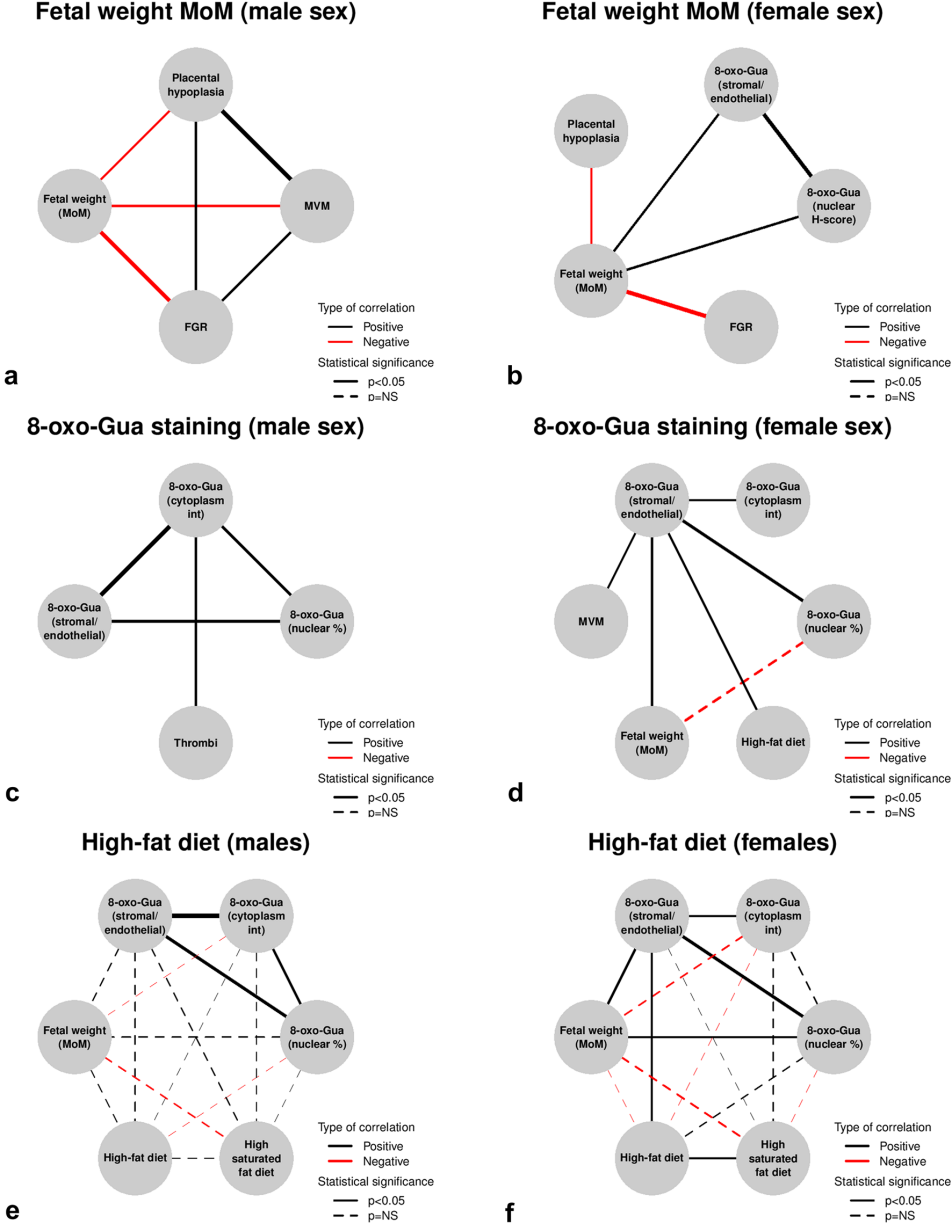
Plot showing correlations assessed with the Spearman test. **a** Correlations between fetal weight, expressed in MoM, and different parameters analyzed among the male fetal population (only rho > 0.2 are shown). **b** Correlations between fetal weight expressed in MoM and other parameters analyzed in the female fetal population (only rho > 0.2 are shown). **c** Correlations between the 8-oxo-Gua staining scores and different parameters analyzed in the male sex (only rho > 0.2 are shown). **d** Correlations between the 8-oxo-Gua staining scores and different parameters analyzed in the female sex (only rho > 0.2 are shown). **e** Correlations between the high-fat diet and other parameters analyzed in the female sex (all correlations shown). **f** Correlations between the high-fat diet and different parameters analyzed in the male sex (all correlations shown). *MVM* maternal vascular malperfusion, *FGR* fetal growth restriction

We then assessed the correlations between the neonatal weight expressed in MoM and the different parameters considered, stratifying by sex. Figure 2a shows the significant correlations with male weight MoM (*p* < 0.05). Figure 2b shows the presence of a significant correlation between increased neonatal weight MoM and increased immunostaining of syncytiotrophoblast nuclei (H-score) and stromal and endothelial cells (intensity score) (*p* < 0.05). Figure 2c shows the correlations between different parameters and immunostaining scores in males. There is a significant correlation between the cytoplasmic staining of syncytiotrophoblast and thrombi formation in the chorionic plate or villi (Fig. 2c) (*p* < 0.05). In females, there is a significant correlation between fetal weight gain and the intensity of immunostaining in stromal and endothelial cells, as shown in Fig. 2d. At the same time, there is a correlation between an increased prevalence of 8-oxo-Gua positive nuclei in the syncytiotrophoblast and reduced MoM of fetal weight. Finally, a significant correlation exists between a high-fat diet and increased immunoreactivity in stromal and endothelial cells (*p* < 0.05). We then correlated the high-fat diet with different variables, starting from the female fetal population. Figure 2e shows a significant positive correlation between a high-fat diet and a high intensity of stromal/endothelial staining (*p* < 0.05). Increased stromal/endothelial staining intensity is also directly related to the fetal weight MoM (*p* < 0.05). In contrast, there are indirectly proportional and non-significant correlations between a diet enriched in fat or saturated fat and a reduction in neonatal weight MoM. In the case of a high saturated fat diet, the correlation reaches a level close to significance (rho = −0.18, *p* = 0.092). In Fig. 2f, the same correlation network was examined in the fetal male population, but the correlations with diet lost significance. Of note, a negative correlation persists between the MoM of fetal weight and a diet rich in saturated fat (rho = −0.14, *p* = 0.224). In addition, although not significant, there are positive correlations between a diet rich in saturated fat and distal villous hypoplasia (rho = 0.22, *p* = 0.052) and accelerated villous maturation (rho = 0.19, *p* = 0.096).

## Discussion

### Main results

This study showed a sex-specific pattern of 8-oxo-Gua staining in placentae of single-term pregnancies, with more oxidative damage found in the nuclei of syncytiotrophoblast cells and stromal and endothelial cells among AGA males compared to AGA females. Second, we found a sex difference in the histological pattern among the late FGR placentae. Finally, male newborns showed a significant correlation between high-intensity 8-oxo-Gua staining in the cytoplasm of syncytiotrophoblast cells and thrombi in the chorionic plate or villi. In contrast, female fetuses had a significant correlation between high-intensity 8-oxo-Gua staining in endothelial and stromal cells and high values of birthweight MoM.

### Results in the context of what is known

#### Sex-based disparities in the immunohistochemical pattern observed in placental tissue

The syncytiotrophoblast, the placenta’s outer layer, comprises fused cells of various ages (Mayhew and Simpson 1994). Because of their proximity to maternal blood and limited antioxidant activity, these cells are vulnerable to reactive oxygen species damage (Cadenas and Davies 2000; Fogarty et al. 2013; Watson et al. 1998). Damaged cells can be identified using the biomarker 8-oxo-Gua (Yoshikawa and Naito 2002). Contrary to our expectations, we did not find a proportional relationship between higher levels of oxidative damage in placentae and decreased fetal growth. However, surprisingly, we found a clear sex-dimorphic pattern of placental oxidative stress for normally grown babies, with males exhibiting significantly higher 8-oxo-Gua staining in syncytiotrophoblast nuclei, as well as stromal and endothelial cells, than females. Syncytiotrophoblast is a steady-state tissue with intracellular components (i.e., intrasyncytial components) that exist between cyto-syncytial fusion and syncytial shedding (Barapatre et al. 2019; Goldman-Wohl and Yagel 2014). Cytotrophoblast cells proliferate, merging into syncytiotrophoblasts and subsequently discharging their senescent nuclei and organelles into the maternal blood (i.e., intrasyncytial phase). Recently, it has been proposed that the rate of progression through the intrasyncytial phase is regulated in a sex-specific manner (Barapatre et al. 2019). Barapatre et al. reported a higher density of non-proliferating nuclei in the villous trophoblast of females rather than males, supporting the idea of a slower progression rate from the cyto-syncytial fusion to the syncytial shedding (Barapatre et al. 2019). Due to the longer permanence in the intrasyncytial phase, the organelles are exposed to increased oxidative damage in females. As opposed to these findings, we observed a higher density of damaged nuclei in syncytiotrophoblast cells in males, suggesting that oxidative stress preferentially marks male placentae. Furthermore, male placentae in our study showed oxidative stress confined to the syncytiotrophoblast and present in stromal and endothelial cells, thus advocating for a broader oxidative stress involvement. This difference between sexes could be attributed to the accelerated growth rates and increased growth outcomes of males. The literature indicates a male-specific preference for growth-signaling pathways throughout gestation (Meakin et al. 2021). In contrast, females prioritize pathways that increase fetoplacental compliance and placental reserve capacity (Meakin et al. 2021).

#### Sex-specific histological pattern observed in FGR placental tissue

Our study reveals that the histological alterations more often reported in placentae of late FGR fetuses are due to MVM, specifically placental hypoplasia and accelerated villous maturation. After stratifying the results by sex, we observed lesions from MVM (accelerated villous maturation) among females and lesions from maternal or fetal malperfusion (avascular villi) among males. Numerous studies have shown that male fetuses are more susceptible to adverse pregnancy conditions, exhibiting a higher incidence of preterm birth, low birth weight, and adverse neonatal outcomes (Ingemarsson 2003; Orzack et al. 2015; Rosa et al. 2019; Zhao et al. 2017). This evidence suggests that male and female fetuses and neonates institute different strategies to cope with an adverse environment. For example, in pregnant women with mild to moderate asthma who did not use inhaled steroids, female fetuses were observed to have a significantly reduced birth weight (Tamayev et al. 2020). In contrast, male birthweights were unaffected (Tamayev et al. 2020). The same trend was observed with preeclampsia, with normal growth trajectories in male fetuses and reduced growth patterns in female fetuses (Murphy et al. 2003; Stark et al. 2006). This evidence suggests that males and females react differently to adversity. Females stop their growth to lower their energy expenditure, whereas males continue to grow normally and are more vulnerable when a second stressful incident happens. As the pregnancy proceeds, female fetuses are more likely to withstand further degradation of the intrauterine environment regarding nutrition and oxygen supply. Recently Chatterjie et al. published the first study on the link between sex-specific human placenta transcriptome and SGA outcomes. The authors found that distinct molecular pathways are involved in the etiological mechanisms of SGA, with male-specific expressed genes correlated with pathways of immune response and inflammation and female-specific expressed genes linked with cellular/organ growth and development (Chatterjee et al. 2022). In this scenario, it might be plausible to observe sex differences even in placental lesion patterns. Our investigation reveals a striking contrast between male and female fetuses with late FGR. Specifically, maternal and fetal malperfusion lesions are more frequently observed in male FGR cases, whereas only maternal malperfusion lesions are evident in female FGR cases. This disparity may be linked to the relatively superior health of female fetal placentae in situations of mild to moderate nutrient and oxygen deprivation, as is typical in late FGR. Notably, our findings confirm that reduced placental reserve capacity in males is a crucial factor in their heightened susceptibility to intrauterine complications and mortality when exposed to unfavorable maternal conditions compared to their female counterparts.

#### Sex-specific correlations between immunohistochemistry and histology

We saw a significant correlation between high 8-oxo-Gua cytoplasmic immunoreactivity in syncytiotrophoblast and chorionic plate or villous thrombi among late male FGR and between high 8-oxo-Gua immunoreactivity in endothelial and stromal cells and high values of birthweight MoM among females. Chorionic plate or villous thrombi originate from FVM and might increase oxidative stress. However, according to recent evidence, the human placenta seems to control even the antioxidant capacity in a sexually dimorphic manner (Muralimanoharan et al. 2015). For example, it has been described that the placentae of male fetuses in obese mothers have a significant decrease in enzymatic antioxidants (Evans and Myatt 2017). If confirmed in non-obese mothers, this mechanism and the hypoxia generated by villous thrombi might explain the increased oxidative damage found in the male syncytiotrophoblast’s cytoplasm. There is growing evidence that females preferentially express glucocorticoid-mediated pathways that permit them to improve their placental reserve capacity to better cope with insults but at the expense of a reduced growth trajectory. In males, the growth trajectory is sustained by androgen-mediated pathways, increasing their intrauterine morbidity and mortality risk.

A recent study looked at the transcripts of cytotrophoblast, syncytiotrophoblast, and arterial and venous endothelial cells isolated from male and female placentas. This research group found sex differentially affected gene expression among all cell phenotypes. More specifically, transcripts of male fetuses prevailed in the epithelial compartment, represented by cytotrophoblast and syncytiotrophoblast. In contrast, arterial and venous endothelial cells showed more female-biased genes. This effect might explain the higher oxidative damage found among endothelial cells in female placentae as far as the female fetus is growing more (Cvitic et al. 2013).

Another phenomenon that could be related to sex-specific patterns of fetal growth is placental genomic imprinting. Genomic imprinting is the fine-tuning of the gene expression dosage in mammals. It consists of an exclusive expression of a gene’s paternally or maternally derived allele, while the other allele is silenced through epigenetic mechanisms (Monk 2015). Interestingly, genomic imprinting is thought to have evolved simultaneously with placentation. Since the genes of paternal origin promote fetal growth and the genes of maternal origin preserve maternal energies, genome imprinting maintains the balance between these two opposed forces, allowing the proper delivery of maternal resources to the developing fetus. Knockout studies of several paternally imprinted genes resulted in fetal growth restriction and smaller placental size. Opposite knockout studies of maternally imprinted genes lead to overgrowth and hyperplasia of the placenta (Frank et al. 2002; Koukoura et al. 2012; Takahashi et al. 2000). Further studies confirmed that genome imprinting is altered in placentae of FGR pregnancies (Diplas et al. 2009; McMinn et al. 2006). In light of this evidence, it could be argued that genomic imprinting is altered differently in males and females, contributing to the differences in histology and immunohistochemistry among small fetuses in our study.

### Clinical implications

Our data provide evidence that sex is a biological factor that influences fetal intrauterine growth and ultimately birth weight. Together with the observation that males weigh ≥ 140 g at term than females, these data suggest the usefulness of customized fetal growth charts in antenatal ultrasound monitoring (Melamed et al. 2013). Of note, recent French research evaluating the different performance between unisex and sex-specific estimated fetal weight charts during third trimester ultrasound in detecting SGA newborns found that using sex-specific charts significantly reduced sex bias in intrauterine growth screening (Monier et al. 2022). Prospective studies on the effect of specific charts rather than unisex charts are warranted to clarify this critical issue, which impacts daily clinical practice.

### Implications for research

Future studies should assess the gene expression in the placenta to understand the main cellular pathways involved in fetal growth under optimal and compromised conditions in terms of nutrient and oxygen delivery to the fetus. Moreover, when these studies are carried out, the need to stratify according to fetal gender should be considered.

### Strengths and limitations

Two main strengths could be recognized in this study. First, the gross examination and histological sampling of the placenta were accurately performed in a standardized manner, according to the recommendations included in the Amsterdam Placental Workshop Group Consensus Statement (Khong et al. 2016). Second, TMA increased tissue resource utilization by allowing tissue from conventional histological paraffin blocks to be relocated so that tissue from multiple blocks could be seen on the same slide.

The main limitation is the low number of late FGR, SGA, and LGA neonates compared to AGA after stratification according to fetal sex. However, the study was sufficiently powered to assess the differences between FGR and AGA in the entire cohort as well as the differences between fetal sexes.

## Conclusions

Our data suggest that oxidative stress may not be the only pathway involved in the pathophysiology of fetal smallness at the gestation term. Furthermore, our immunohistochemical and histological findings revealed an unexpected difference in the oxidative stress pattern between male and female placentae, suggesting that fetal growth is differently regulated between the two sexes.

### Supplementary Information

Below is the link to the electronic supplementary material.

Supplementary file1 (TIF 120 KB)

Supplementary file2 (DOCX 26 KB)

## Data Availability

The data supporting the findings of this study are available, but they are not publicly accessible because they were used under license and are therefore subject to restrictions. However, data are available from the authors upon reasonable request and approval from the Ethics Committee.

## Abbreviations

8-oxo-Gua: 8-Hydroxyguanine
AGA: Appropriate for gestational age
BMI: Body mass index
FFQ: Food frequency questionnaire
FGR: Fetal growth restriction
FMV: Fetal vascular malperfusion
IG-21: Intergrowth 21
IHC: Immunohistochemistry
IQR: Interquartile range
LGA: Large for gestational age
MoM: Multiple of the median
MVM: Maternal vascular malperfusion
ROS: Reactive oxygen species
SGA: Small for gestational age
TMA: Tissue microarray
