# Supplementary figures and images for: Fetal growth at term and placental oxidative stress in a tissue micro-array model: a histological and immunohistochemistry study
Source: Histochem Cell Biol. 2023 Jun 12;160(4):293–306. doi: 10.1007/s00418-023-02212-6 (PMC10509069; doi:10.1007/s00418-023-02212-6)

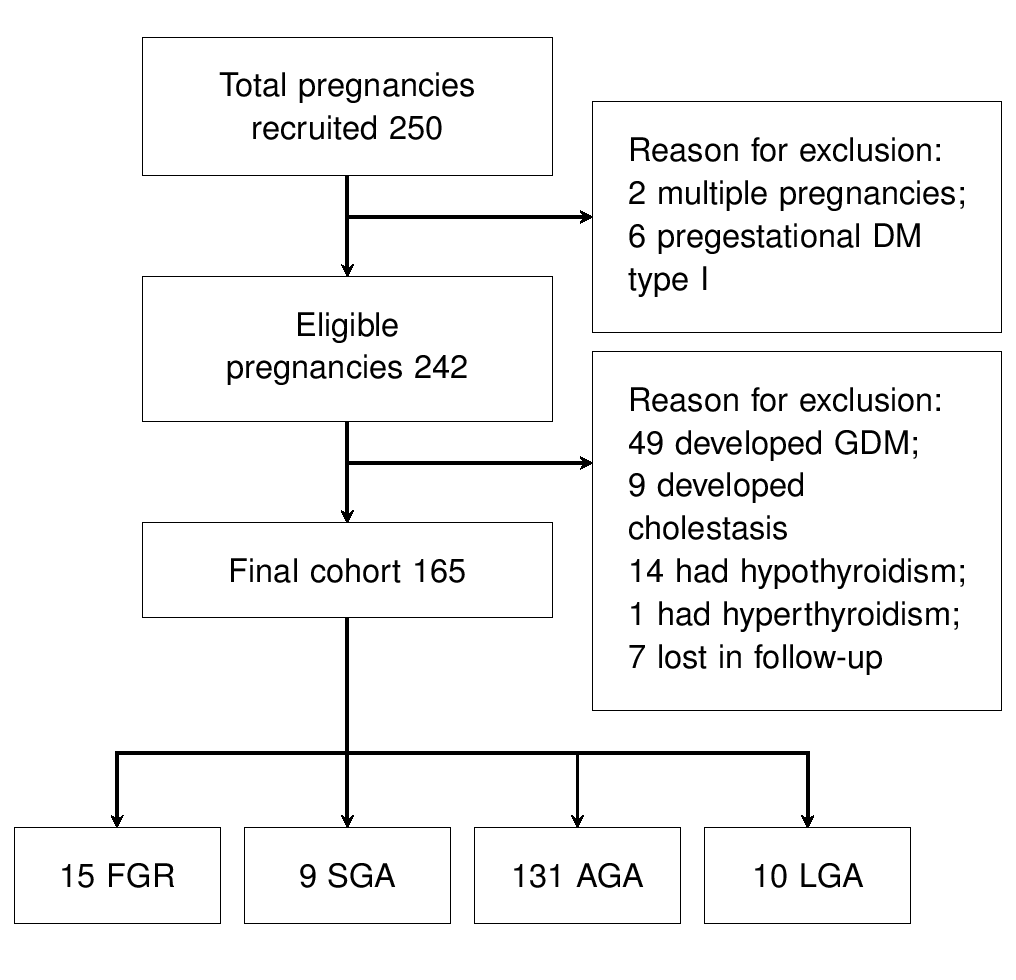

Supplement: Supplementary file 1 — Supplementary file1 (TIF 120 KB) [file 418_2023_2212_MOESM1_ESM.tif]
