# Supplementary material for: Fetal growth at term and placental oxidative stress in a tissue micro-array model: a histological and immunohistochemistry study
Source: Histochem Cell Biol. 2023 Jun 12;160(4):293–306. doi: 10.1007/s00418-023-02212-6 (PMC10509069; doi:10.1007/s00418-023-02212-6)
Supplement: Supplementary file 2 — Supplementary file2 (DOCX 26 KB) [file 418_2023_2212_MOESM2_ESM.docx]

**Supplementary Table 1**- Population description.

| Maternal characteristics |  |
| --- | --- |
| Maternal age (years) | 33 (30-36) |
| Nulliparity | 53.33% (88/165) |
| Pre-pregnancy BMI (kg/m²) | 22.95 (19.92-26.45) |
| Region of origin |  |
| Italy | 84.24% (139/165) |
| Europe and Central Asia | 9.7% (16/165) |
| Sub-Saharan Africa | 3.03% (5/165) |
| Middle East and North Africa | 1.82% (3/165) |
| South Asia | 1.21% (2/165) |
| East Asia and Pacific | 0% (0/165) |
| Latin America and Caribbean | 0% (0/165) |
| Pregnancy characteristics |  |
| Tobacco smoke in pregnancy | 6.06% (10/165) |
| Labor mode |  |
| Spontaneous | 31.52% (52/165) |
| Labor induction | 55.76% (92/165) |
| Without labor | 12.73% (21/165) |
| Delivery mode |  |
| Spontaneous vaginal delivery | 61.82% (102/165) |
| Vacuum extraction | 16.36% (27/165) |
| Cesarean delivery | 21.82% (36/165) |
| Neonatal characteristics |  |
| Male sex | 47.27% (78/165) |
| Gestational age (weeks) | 39 (38-40) |
| Neonatal weight (g) | 3277.85 (±513.52) |
| Neonatal weight (MoM) | 0.99 (±0.13) |
| Placental index | 0.13 (0.12-0.15) |
| Placental weight (MoM) | 1 (0.84-1.15) |
| Fetal growth |  |
| FGR | 9.09% (15/165) |
| SGA | 5.45% (9/165) |
| AGA | 79.39% (131/165) |
| LGA | 6.06% (10/165) |
| Apgar score 1st minute | 8 (8-9) |
| Apgar score 5th minute | 9 (9-10) |
| Cord blood pH | 7.28 (7.23-7.32) |
| Base excess | 3 (2-6) |
| NICU hospitalization | 6.06% (10/165) |
| Neonatal resucitation | 10.3% (17/165) |

**Supplementary Table 2**- Placental histology assessed according to the Amsterdam criteria.

| Histology |  |
| --- | --- |
| Maternal vascular malperfusion | 24.24% (40/165) |
| Placental hypoplasia | 9.09% (15/165) |
| Placental infarction | 10.91% (18/165) |
| Retroplacental hemorrhage | 1.21% (2/165) |
| Accelerated villous maturation | 2.42% (4/165) |
| Distal villous hypoplasia | 4.24% (7/165) |
| Fetal vascular malperfusion | 12.12% (20/165) |
| Avascular villi | 5.45% (9/165) |
| Thrombi (chorionic plate or major stem villi) | 8.48% (14/165) |
| Delayed villous maturation | 60.61% (100/165) |

**Supplementary Table 3**- Placental immunohistochemical (IHC) 8-hydroxyguanine (8-oxo-Gua) staining.

| IHC |  |
| --- | --- |
| Nuclear staining syncytiotrophoblast (H-score) | 65.00 (35.00-113.12) |
| Nuclear staining syncytiotrophoblast (intensity score) | 2.00 (2.00-2.50) |
| Nuclear staining syncytiotrophoblast (Percentage of positive nuclei) | 30.00 (17.50-50.62) |
| Cytoplasm staining syncytiotrophoblast (intensity score) | 1.50 (1.00-2.00) |
| Stromal and endothelial cells (intensity score) | 2.50 (2.00-3.00) |

**Supplementary Table 4**- Population characteristics differences between fetal growth groups.

|  | FGR (15) | SGA (9) | AGA (131) | LGA (10) | p |
| --- | --- | --- | --- | --- | --- |
| Maternal characteristics |  |  |  |  |  |
| Maternal age (years) | 33.00 (30.00-34.50) | 37.00 (31.00-39.00) | 33.00 (30.00-36.00) | 31.50 (29.00-34.75) | NS |
| Nulliparity | 60.00% (9/15) | 66.67% (6/9) | 53.44% (70/131) | 30.00% (3/10) | NS |
| Pre-pregnancy BMI (kg/m²) | 19.10 (17.99-22.66) | 22.04 (18.78-24.39) | 23.31 (20.31-27.06) | 21.91 (19.53-24.96) | 2 |
| Region of origin |  |  |  |  | 0.880* |
| Italy | 73.33% (11/15) | 77.78% (7/9) | 85.50% (112/131) | 90.00% (9/10) | NS |
| Europe and Central Asia | 20.00% (3/15) | 11.11% (1/9) | 8.40% (11/131) | 10.00% (1/10) | NS |
| Sub-Saharan Africa | 6.67% (1/15) | 11.11% (1/9) | 2.29% (3/131) | 0.00% (0/10) | NS |
| Middle East and North Africa | 0.00% (0/15) | 0.00% (0/9) | 2.29% (3/131) | 0.00% (0/10) | NS |
| South Asia | 0.00% (0/15) | 0.00% (0/9) | 1.53% (2/131) | 0.00% (0/10) | NS |
| Pregnancy characteristics |  |  |  |  |  |
| Tobacco smoke in pregnancy | 6.67% (1/15) | 11.11% (1/9) | 6.11% (8/131) | 0.00% (0/10) | NS |
| Labor mode |  |  |  |  | 0.558* |
| Spontaneous | 20.00% (3/15) | 22.22% (2/9) | 32.06% (42/131) | 50.00% (5/10) | NS |
| Labor induction | 66.67% (10/15) | 77.78% (7/9) | 54.20% (71/131) | 40.00% (4/10) | NS |
| Without labor | 13.33% (2/15) | 0.00% (0/9) | 13.74% (18/131) | 10.00% (1/10) | NS |
| Delivery mode |  |  |  |  | 0.366* |
| Spontaneous vaginal delivery | 60.00% (9/15) | 88.89% (8/9) | 58.78% (77/131) | 80.00% (8/10) | NS |
| Vacuum extraction | 26.67% (4/15) | 0.00% (0/9) | 16.79% (22/131) | 10.00% (1/10) | NS |
| Cesarean delivery | 13.33% (2/15) | 11.11% (1/9) | 24.43% (32/131) | 10.00% (1/10) | NS |
| Neonatal characteristics |  |  |  |  |  |
| Male sex | 40.00% (6/15) | 77.78% (7/9) | 45.04% (59/131) | 60.00% (6/10) | NS |
| Gestational age (weeks) | 38.00 (37.00-39.50) | 40.00 (39.00-40.00) | 39.00 (38.00-40.00) | 39.00 (39.00-39.00) | NS |
| Neonatal weight (g) | 2496.00 (2252.50-2613.00) | 2900.00 (2700.00-3070.00) | 3355.00 (3102.50-3637.50) | 4096.00 (4007.50-4240.00) | 1,2,3,4,5,6 |
| Neonatal weight (MoM) | 0.78 (0.73-0.80) | 0.84 (0.81-0.86) | 1.01 (0.93-1.08) | 1.23 (1.22-1.28) | 1,2,3,4,5,6 |
| Placental index | 0.14 (0.12-0.16) | 0.13 (0.12-0.15) | 0.13 (0.12-0.15) | 0.13 (0.12-0.16) | NS |
| Placental weight (MoM) | 0.82 (0.72-0.89) | 0.88 (0.82-0.97) | 1.00 (0.88-1.13) | 1.19 (1.12-1.46) | 2,3,5,6 |
| Apgar score 1st minute | 9.00 (7.50-9.00) | 8.00 (8.00-9.00) | 8.00 (8.00-9.00) | 8.50 (8.00-9.00) | NS |
| Apgar score 5th minute | 9.00 (9.00-9.00) | 9.00 (9.00-9.00) | 9.00 (9.00-10.00) | 9.00 (9.00-9.75) | NS |
| Cord blood pH | 7.30 (7.26-7.31) | 7.25 (7.22-7.31) | 7.27 (7.23-7.32) | 7.29 (7.23-7.32) | NS |
| Base excess | 3.00 (2.00-6.50) | 5.00 (3.00-9.00) | 3.50 (2.00-6.00) | 2.00 (0.00-3.00) | NS |
| NICU hospitalization | 6.67% (1/15) | 0.00% (0/9) | 6.11% (8/131) | 10.00% (1/10) | NS |
| Neonatal resucitation | 6.67% (1/15) | 0.00% (0/9) | 11.45% (15/131) | 10.00% (1/10) | NS |

Differences statistically significant (p<0.05): 1) FGR v.s. SGA; 2) FGR v.s. AGA; 3) FGR v.s. LGA; 4) SGA v.s. AGA; 5) SGA v.s. LGA; 6) AGA v.s. LGA.

**Supplementary Table 5**- Population characteristics differences between fetal males and females in AGA newborns.

|  | M (59) | F (72) | p |
| --- | --- | --- | --- |
| Maternal characteristics |  |  |  |
| Maternal age (years) | 32.00 (29.50-35.50) | 34.00 (30.00-36.00) | 0.241 |
| Nulliparity | 45.76% (27/59) | 59.72% (43/72) | 0.111 |
| Pre-pregnancy BMI (kg/m²) | 23.18 (20.20-26.81) | 23.34 (20.40-27.09) | 0.982 |
| Region of origin |  |  |  |
| Italy | 91.53% (54/59) | 80.56% (58/72) | 0.076 |
| Europe and Central Asia | 3.39% (2/59) | 12.50% (9/72) | 0.061 |
| Middle East and North Africa | 3.39% (2/59) | 1.39% (1/72) | 0.446 |
| Sub-Saharan Africa | 0.00% (0/59) | 4.17% (3/72) | 0.113 |
| South Asia | 1.69% (1/59) | 1.39% (1/72) | 0.887 |
| Pregnancy characteristics |  |  |  |
| Tobacco smoke in pregnancy | 5.08% (3/59) | 6.94% (5/72) | 0.658 |
| Labor mode |  |  | 0.137 |
| Spontaneous | 28.81% (17/59) | 34.72% (25/72) | 0.471 |
| Labor induction | 50.85% (30/59) | 56.94% (41/72) | 0.486 |
| Without labor | 20.34% (12/59) | 8.33% (6/72) | <0.05 |
| Delivery mode |  |  | 0.341 |
| Spontaneous vaginal delivery | 54.24% (32/59) | 62.50% (45/72) | 0.339 |
| Vacuum extraction | 15.25% (9/59) | 18.06% (13/72) | 0.670 |
| Cesarean delivery | 30.51% (18/59) | 19.44% (14/72) | 0.143 |
| Neonatal characteristics |  |  |  |
| Male sex | 100.00% (59/59) | 0.00% (0/72) | <0.05 |
| Gestational age (weeks) | 39.00 (39.00-40.00) | 39.00 (38.00-40.00) | 0.485 |
| Neonatal weight (g) | 3515.00 (3185.00-3685.00) | 3248.00 (3022.50-3462.50) | <0.05 |
| Neonatal weight (MoM) | 1.02 (0.97-1.09) | 1.00 (0.92-1.06) | 0.055 |
| Placental index | 0.12 (0.11-0.14) | 0.14 (0.13-0.16) | <0.05 |
| Placental weight (MoM) | 1.00 (0.88-1.10) | 1.02 (0.87-1.13) | 0.664 |
| Apgar score 1st minute | 8.00 (8.00-9.00) | 8.00 (7.00-9.00) | 0.671 |
| Apgar score 5th minute | 9.00 (9.00-9.50) | 9.00 (9.00-10.00) | 0.669 |
| Apgar score 1st minute | 8.00 (8.00-9.00) | 8.00 (7.00-9.00) | 0.671 |
| Apgar score 5th minute | 9.00 (9.00-9.50) | 9.00 (9.00-10.00) | 0.669 |
| NICU hospitalization | 1.69% (1/59) | 9.72% (7/72) | 0.056 |
| Neonatal resucitation | 8.47% (5/59) | 13.89% (10/72) | 0.333 |

**Supplementary Table 6**- Population characteristics differences between fetal growth groups. In this analysis, only female fetuses were included.

|  | FGR (9) | SGA (2) | AGA (72) | LGA (4) | p |
| --- | --- | --- | --- | --- | --- |
| Maternal characteristics |  |  |  |  |  |
| Maternal age (years) | 31.00 (26.00-35.00) | 41.00 (39.00-43.00) | 34.00 (30.00-36.00) | 30.50 (29.00-32.50) | NS |
| Nulliparity | 66.67% (6/9) | 50.00% (1/2) | 59.72% (43/72) | 25.00% (1/4) | NS |
| Pre-pregnancy BMI (kg/m²) | 19.41 (18.23-21.66) | 20.41 (19.59-21.23) | 23.34 (20.40-27.09) | 23.65 (22.25-24.87) | 2 |
| Pregnancy characteristics |  |  |  |  |  |
| Tobacco smoke in pregnancy | 0.00% (0/9) | 0.00% (0/2) | 6.94% (5/72) | 0.00% (0/4) | NS |
| Labor mode |  |  |  |  |  |
| Spontaneous | 33.33% (3/9) | 50.00% (1/2) | 34.72% (25/72) | 50.00% (2/4) | NS |
| Labor induction | 66.67% (6/9) | 50.00% (1/2) | 56.94% (41/72) | 50.00% (2/4) | NS |
| Without labor | 0.00% (0/9) | 0.00% (0/2) | 8.33% (6/72) | 0.00% (0/4) | NS |
| Delivery mode |  |  |  |  |  |
| Spontaneous vaginal delivery | 77.78% (7/9) | 100.00% (2/2) | 62.50% (45/72) | 100.00% (4/4) | NS |
| Vacuum extraction | 22.22% (2/9) | 0.00% (0/2) | 18.06% (13/72) | 0.00% (0/4) | NS |
| Cesarean delivery | 0.00% (0/9) | 0.00% (0/2) | 19.44% (14/72) | 0.00% (0/4) | NS |
| Neonatal characteristics |  |  |  |  |  |
| Gestational age (weeks) | 39.00 (37.00-40.00) | 39.50 (39.25-39.75) | 39.00 (38.00-40.00) | 39.00 (39.00-39.25) | NS |
| Neonatal weight (g) | 2496.00 (2205.00-2646.00) | 2757.50 (2696.25-2818.75) | 3248.00 (3022.50-3462.50) | 4117.50 (4082.50-4172.25) | 2,3,4,6 |
| Neonatal weight (MoM) | 0.79 (0.74-0.80) | 0.83 (0.82-0.83) | 1.00 (0.92-1.06) | 1.26 (1.23-1.30) | 2,3,4,6 |
| Placental index | 0.14 (0.13-0.17) | 0.13 (0.12-0.14) | 0.14 (0.13-0.16) | 0.14 (0.13-0.15) | NS |
| Placental weight (MoM) | 0.81 (0.76-0.89) | 0.79 (0.75-0.84) | 1.02 (0.87-1.13) | 1.23 (1.21-1.32) | 2,3,6 |
| Apgar score 1st minute | 9.00 (8.00-9.00) | 9.00 (9.00-9.00) | 8.00 (7.00-9.00) | 9.00 (8.75-9.00) | NS |
| Apgar score 5th minute | 9.00 (9.00-9.00) | 9.00 (9.00-9.00) | 9.00 (9.00-10.00) | 9.50 (9.00-10.00) | NS |
| Apgar score 1st minute | 9.00 (8.00-9.00) | 9.00 (9.00-9.00) | 8.00 (7.00-9.00) | 9.00 (8.75-9.00) | NS |
| Apgar score 5th minute | 9.00 (9.00-9.00) | 9.00 (9.00-9.00) | 9.00 (9.00-10.00) | 9.50 (9.00-10.00) | NS |
| NICU hospitalization | 0.00% (0/9) | 0.00% (0/2) | 9.72% (7/72) | 0.00% (0/4) | NS |
| Neonatal resucitation | 0.00% (0/9) | 0.00% (0/2) | 13.89% (10/72) | 0.00% (0/4) | NS |

Differences statistically significant (p<0.05): 1) FGR v.s. SGA; 2) FGR v.s. AGA; 3) FGR v.s. LGA; 4)

**Supplementary Table 7**- Population characteristics differences between fetal growth groups. In this analysis, only male fetuses were included.

|  | FGR (6) | SGA (7) | AGA (59) | LGA (6) | p |
| --- | --- | --- | --- | --- | --- |
| Maternal characteristics |  |  |  |  |  |
| Maternal age (years) | 33.50 (33.00-34.00) | 33.00 (30.50-38.50) | 32.00 (29.50-35.50) | 33.00 (27.25-35.75) | NS |
| Nulliparity | 50.00% (3/6) | 71.43% (5/7) | 45.76% (27/59) | 33.33% (2/6) | NS |
| Pre-pregnancy BMI (kg/m²) | 18.82 (17.63-22.66) | 24.11 (20.18-25.17) | 23.18 (20.20-26.81) | 20.09 (18.99-24.09) | NS |
| Pregnancy characteristics |  |  |  |  |  |
| Tobacco smoke in pregnancy | 16.67% (1/6) | 14.29% (1/7) | 5.08% (3/59) | 0.00% (0/6) | NS |
| Labor mode |  |  |  |  |  |
| Spontaneous | 0.00% (0/6) | 14.29% (1/7) | 28.81% (17/59) | 50.00% (3/6) | 3 |
| Labor induction/augmentation | 66.67% (4/6) | 85.71% (6/7) | 50.85% (30/59) | 33.33% (2/6) | NS |
| Without labor | 33.33% (2/6) | 0.00% (0/7) | 20.34% (12/59) | 16.67% (1/6) | NS |
| Deliery mode |  |  |  |  |  |
| Spontaneous vaginal delivery | 33.33% (2/6) | 85.71% (6/7) | 54.24% (32/59) | 66.67% (4/6) | NS |
| Vacum extraction | 33.33% (2/6) | 0.00% (0/7) | 15.25% (9/59) | 16.67% (1/6) | NS |
| Cesarean delivery | 33.33% (2/6) | 14.29% (1/7) | 30.51% (18/59) | 16.67% (1/6) | NS |
| Neonatal characteristics |  |  |  |  |  |
| Gestational age (weeks) | 37.50 (37.00-38.75) | 40.00 (39.00-40.00) | 39.00 (39.00-40.00) | 39.00 (39.00-39.00) | NS |
| Neonatal weight (g) | 2427.50 (2312.50-2557.50) | 3025.00 (2800.00-3072.50) | 3515.00 (3185.00-3685.00) | 4046.00 (3943.75-4229.25) | 1,2,3,4,5,6 |
| Neonatal weight (MoM) | 0.76 (0.73-0.78) | 0.85 (0.80-0.86) | 1.02 (0.97-1.09) | 1.22 (1.17-1.26) | 1,2,3,4,5,6 |
| Placental index | 0.14 (0.12-0.16) | 0.13 (0.12-0.16) | 0.12 (0.11-0.14) | 0.12 (0.11-0.15) | NS |
| Placental weight (MoM) | 0.83 (0.72-0.96) | 0.92 (0.83-1.04) | 1.00 (0.88-1.10) | 1.13 (1.09-1.43) | 3 |
| Apgar score 1st minute | 8.50 (7.25-9.00) | 8.00 (8.00-8.50) | 8.00 (8.00-9.00) | 8.00 (8.00-8.75) | NS |
| Apgar score 5th minute | 9.00 (8.25-9.00) | 9.00 (9.00-9.00) | 9.00 (9.00-9.50) | 9.00 (8.25-9.00) | NS |
| Apgar score 1st minute | 8.50 (7.25-9.00) | 8.00 (8.00-8.50) | 8.00 (8.00-9.00) | 8.00 (8.00-8.75) | NS |
| Apgar score 5th minute | 9.00 (8.25-9.00) | 9.00 (9.00-9.00) | 9.00 (9.00-9.50) | 9.00 (8.25-9.00) | NS |
| NICU hospitalization | 16.67% (1/6) | 0.00% (0/7) | 1.69% (1/59) | 16.67% (1/6) | 2,6 |
| Neonatal resucitation | 16.67% (1/6) | 0.00% (0/7) | 8.47% (5/59) | 16.67% (1/6) | NS |

Differences statistically significant (p<0.05): 1) FGR v.s. SGA; 2) FGR v.s. AGA; 3) FGR v.s. LGA; 4) SGA v.s. AGA; 5) SGA v.s. LGA; 6) AGA v.s. LGA.
